# Complete Genome Sequence of a Newly Emerging Newcastle Disease Virus Isolated in China

**DOI:** 10.1128/genomeA.00169-13

**Published:** 2013-05-09

**Authors:** Minhua Sun, Jiawen Dong, Zhaoxiong Wang, Linlin Li, Jianfeng Yuan, Peirong Jiao, Qilin Hu, Tao Ren

**Affiliations:** Key Laboratory of Animal Disease Control and Prevention of the Ministry of Agriculture, College of Veterinary Medicine, South China Agricultural University, Guangzhou, Guangdong, People’s Republic of Chinaa;; Guangdong Open Laboratory of Veterinary Public Health, Institute of Animal Health, Guangdong Academy of Agricultural Sciences, Tianhe, Guangzhou, Guangdong, People’s Republic of Chinab;; College of Animal Science, Yangtze University, Jingzhou, Hubei, People’s Republic of Chinac

## Abstract

Goose/GD/2010 is a newly emerging Newcastle disease virus (NDV) isolated from a sick goose flock in southern China. Here, we report the complete genome sequence of this isolate, which belongs to NDV subgenotype VIIb. This is the first report about the complete genome information of an isolate of subgenotype VIIb in China.

## GENOME ANNOUNCEMENT

Newcastle disease (ND) is a constant threat to domestic birds (1). Its causative agent is Newcastle disease virus (NDV). NDV has a single serotype; however, it can be genetically divided into two classes, class I and class II, both of which can be further categorized into at least 10 genotypes (2–6). To date, viruses in class II have been responsible for all four ND pandemics since 1926 (7). In the later 1990s, genotype VII viruses, which caused the fourth ND pandemic, have been mostly isolated in Asian countries (2, 3, 7). Genotype VII can be further divided into at least six subgenotypes (VIIa to VIIf) (2, 7), although the subgenotype VIIb had not yet been reported in China.

In this study, a newly emerging NDV isolate, Goose/GD/2010, was isolated in a goose flock in southern China and caused about 30% mortality. The complete sequence of Goose/GD/2010 was amplified by eight pairs of oligonucleotide primers and was subsequently cloned into the pMD18-T vector (TaKaRa, Japan), followed by sequencing with an ABI3730 genome sequencer.

The complete genome sequence of Goose/GD/2010 is 15,192 nucleotides (nt) in length, with a G+C content of 46.04%. It comprises six genes in the order of 3′–NP–P–M–F–HN–L–5′. Similar to other NDV isolates, the 3′ leader and 5′ trailer sequences of Goose/GD/2010 consist of 55 and 114 nt, respectively. The intergenic sequences (IGSs) were also conserved. There was only 1 nt each in the NP–P, P–M, and M–F gene junctions, while 31 nt and 47 nt were found in the F–HN and HN–L gene junctions, respectively. A six-nucleotide insertion (CCCTAC), shared by genotypes V to VIII (8), was also observed after nt position 1647 in the 5′ noncoding region of the nucleoprotein (NP) gene. The fusion (F) protein cleavage site, as the molecular basis for pathogenicity (9–11), was ^112^RRQKRF^117^, which indicated that Goose/GD/2010 is velogenic (9–11). The seven neutralizing epitopes on the F protein (12) are highly conserved, whereas the antigenic site I on the hemagglutinin-neuraminidase (HN) protein (13), also known as a variable epitope (14), harbored an E347D mutation that might be caused under vaccination pressure (14).

Phylogenetic analysis indicated that Goose/GD/2010 can be classified into NDV subgenotype VIIb. Genome sequence analysis showed that Goose/GD/2010 shares 99% nucleotide identity with the strains GD1003/2010 (GenBank accession no. KC152049) and GD450/2011 (GenBank accession no. KC152048), which means they probably come from the same outbreak with a common ancestor. Since the 1990s, subgenotype VIIb, which originated in Southeast Asia (15), has caused many outbreaks in southern Africa, Europe, and Asia (7, 16). However, phylogenetic analysis based on the F gene nucleotides showed that Goose/GD/2010 has a higher correlation (>93%) with the early NDV strains BMYBU87078 (GenBank accession no. AY175677) and MB076/05 (GenBank accession no. GQ901892), which were both isolated in Malaysia (16, 17). For this reason, the newly emerging NDV in China might have originated in Malaysia. Taken together, the findings from this study will be helpful for understanding the evolution of the NDV genome.

### Nucleotide sequence accession number.

The genome sequence of Goose/GD/2010 was deposited in GenBank under the accession no. KC551967.
